# Top-Down Network Effective Connectivity in Abstinent Substance Dependent Individuals

**DOI:** 10.1371/journal.pone.0164818

**Published:** 2016-10-24

**Authors:** Michael F. Regner, Naomi Saenz, Keeran Maharajh, Dorothy J. Yamamoto, Brianne Mohl, Korey Wylie, Jason Tregellas, Jody Tanabe

**Affiliations:** 1 Department of Radiology, University of Colorado School of Medicine, Aurora, CO, 80045, United States of America; 2 Department of Bioengineering, University of Colorado College of Engineering and Applied Sciences, Aurora, CO, 80045, United States of America; 3 Department of Psychiatry, University of Colorado School of Medicine, Aurora, CO, 80045, United States of America; University of Cambridge, UNITED KINGDOM

## Abstract

**Objective:**

We hypothesized that compared to healthy controls, long-term abstinent substance dependent individuals (SDI) will differ in their effective connectivity between large-scale brain networks and demonstrate increased directional information from executive control to interoception-, reward-, and habit-related networks. In addition, using graph theory to compare network efficiencies we predicted decreased small-worldness in SDI compared to controls.

**Methods:**

50 SDI and 50 controls of similar sex and age completed psychological surveys and resting state fMRI. fMRI results were analyzed using group independent component analysis; 14 networks-of-interest (NOI) were selected using template matching to a canonical set of resting state networks. The number, direction, and strength of connections between NOI were analyzed with Granger Causality. Within-group thresholds were *p*<0.005 using a bootstrap permutation. Between group thresholds were *p*<0.05, FDR-corrected for multiple comparisons. NOI were correlated with behavioral measures, and group-level graph theory measures were compared.

**Results:**

Compared to controls, SDI showed significantly greater Granger causal connectivity from right executive control network (RECN) to dorsal default mode network (dDMN) and from dDMN to basal ganglia network (BGN). RECN was negatively correlated with impulsivity, behavioral approach, and negative affect; dDMN was positively correlated with impulsivity. Among the 14 NOI, SDI showed greater bidirectional connectivity; controls showed more unidirectional connectivity. SDI demonstrated greater global efficiency and lower local efficiency.

**Conclusions:**

Increased effective connectivity in long-term abstinent drug users may reflect improved cognitive control over habit and reward processes. Higher global and lower local efficiency across all networks in SDI compared to controls may reflect connectivity changes associated with drug dependence or remission and requires future, longitudinal studies to confirm.

## Introduction

Substance dependence is a significant public health problem with an estimated 10.3% lifetime prevalence in the United States [1]. Across substances of abuse, a generalizable pattern develops beginning with an initial stage of rewarding effects from occasional use and developing into a pathologic stage of loss of control, escalated use, compulsive drug seeking, and significant negative consequences [2]. Individuals with substance dependence have been shown to exhibit higher levels of impulsivity, behavioral approach, and negative affect [3], and these differences have been associated with structural [4] and functional [5–8] brain differences compared to healthy controls. While task-based studies using fMRI and PET have contributed significantly to our understanding of functional brain changes in specific neuroanatomical areas [9], resting-state fMRI (rsfMRI) provides opportunity to explore large-scale networks and network interactions independent of task-specific neuropsychological constructs [10]. Advantages of rsfMRI include less confounding by differences in task paradigms, correlation of resting state networks (RSN) to specific tasks and neuropsychiatric constructs [11], and reproducibility due to simplified experimental design and data acquisition [12].

**Fig 1:**
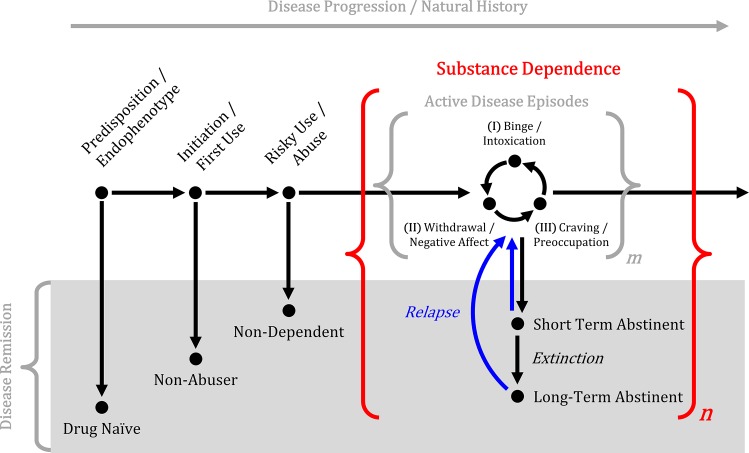
Simplified illustration of the natural history of substance use disorders. Active substance dependence is characterized by sequential stages of binge/intoxication, withdrawal/negative affect, and craving/preoccupation [13] that repeat a variable number of cycles, represented by *m*. Active disease episodes may be interrupted by periods of short- or long-term abstinence, represented by *n*.

Stimulant dependence is characterized by complex behaviors and, like other neuropsychiatric diseases, is thought to reflect pathology at the circuit-level rather than a single brain structure [13]. Moreover, activity and connectivity differences in stimulant dependence have been demonstrated using rsfMRI across disease stages and may explain the progressive behavioral phenotype changes across the natural history of the disorder [14]. For example, active drug addiction stages include (I) binge/intoxication, (II) withdrawal/negative affect, and (III) preoccupation/anticipation [13]; involved circuits at these stages include (I) ventral tegmental area and striatum; (II) amygdala, bed nucleus of the stria terminalis, and ventral striatum; and (III) prefrontal cortex, hippocampus, basolateral amygdala, cingulate, and insula. Sequential cycling through these active disease stages is hypothesized to result in the neuroadaptive changes that give rise to compulsive drug-seeking and drug-taking (**Fig 1**).

Brain activity and connectivity at different disease stages have been correlated with individual differences in executive function, interoception, reward, and habit formation. For example, Gu et al. [15] observed decreased rsfMRI connectivity between nodes within the mesocorticolimbic reward pathway in active cocaine users compared to healthy controls. These findings are consistent with animal models, in which rats dependent upon and self-administering cocaine demonstrated decreased connectivity compared to control rats [16]; affected pathways in this sample of rats included connections between the dorsolateral prefrontal cortex (PFC) and ventral striatum, as well as between the prelimbic cortex (homologous to anterior cingulate gyrus in humans) and entopeduncular nucleus (homologous to globus pallidus interna in humans) [16]. These active disease findings stand in contrast to findings in disease remission. In short-term abstinent cocaine dependence (≥3 days), Wilcox et al. [17] observed increased rsfMRI connectivity between the ventral striatum and ventromedial PFC. Camchong and colleagues [18] measured resting state functional connectivity amongst reward processing regions in a cohort of stimulant dependent individuals at two time points, 5 weeks abstinence and 13 weeks abstinence, with comparison to a matched healthy control group. Abstinent stimulant dependent patients demonstrated increased functional connectivity compared to controls, consistent with prior studies in patients of 1.4 years of abstinence [5] and 5.7 years of abstinence [19]. Although Camchong et al. [18] found abstinent stimulant dependent patients demonstrated increased functional connectivity compared to controls, patients who relapsed between time points demonstrated decreased connectivity compared to patients who maintained abstinence. The authors speculated that this reduction in functional connectivity from 5 to 13 weeks in relapsers compared to abstinent patients may be associated with these patients’ inability to maintain abstinence. These studies suggest that group differences in connectivity may be related to different stages of dependence/remission, possibly representing a transition from hypoconnectivity in limbic and subcortical regions during active dependence to increased top-down executive control in sustained abstinence.

Understanding differences in large-scale brain connectivity depends upon characterizing the relative activity within networks as well as between them. Two modes of functional interactions between brain regions include functional connectivity and effective connectivity. Functional connectivity is the simultaneous and temporally coherent activation of separate brain regions. Effective connectivity characterizes the directional flow of information. One method of characterizing effective connectivity is Granger causality [20], which is methodologically straightforward but requires careful application and interpretation.

To date, no study has investigated the effective connectivity differences in stimulant dependence. This is important because understanding the direction of information flow in large-scale brain networks may further elucidate mechanisms of abstinence and explain previously reported changes. To improve substance dependence treatments, a better understanding of the connectivity characteristics associated with long term remission are needed and may help to predict successful abstinence, evaluate treatment efficacy, and develop novel treatments. This study investigated the effective connectivity and graph theory characteristics of large scale networks in the resting brain in long-term abstinent SDI compared to healthy controls to provide holistic, organ-level measures of brain connectivity and organization for comparisons between groups. We hypothesized that compared to healthy controls, long-term abstinent SDI will demonstrate altered effective connectivity between large-scale brain networks and increased directional information from executive control to interoception-, reward-, and habit-related networks.

## Materials and Methods

### Sample Population

Fifty substance dependent individuals (SDI) and 50 healthy controls matched in age and sex were prospectively recruited between October 2010 and June 2013. Demographic information is reported in **Table 1**. SDI were recruited from a residential treatment program at the University of Colorado Denver Addiction Research Treatment Services. Inclusion criteria for SDI were lifetime DSM-IV psychostimulant dependence (methamphetamine, cocaine, or amphetamine-class substances) and abstinence from all drugs of abuse for a minimum 60 days, verified through close supervision and random urine screens. Participants were permitted to have previously met dependence criteria for substances other than psychostimulants due to the high prevalence of polysubstance use in people dependent upon psychostimulants. Average abstinence duration was 12.8 ± 12.4 months. Healthy controls were recruited from the community and excluded if dependent on alcohol or other drugs of abuse except tobacco. Exclusion criteria for all participants included major depression within the last two months, psychosis, neurological illness, prior head trauma with loss of consciousness exceeding 15 minutes, prior neurosurgery, HIV, bipolar disorder, other major medical illness, inability to tolerate MRI, positive urine or saliva screen (AccuTest^TM^, AlcoScreen^TM^), and IQ < 80. All participants provided written informed consent approved by the Colorado Multiple Institutional Review Board.

**Table 1:** Demographic, drug use, and behavioral characteristics of the sample population.

	SDI	Control	t-value	p-value
***Demographics***				
N	50 (22F/28M)	50 (25F/25M)		0.869
Age	34.18±7.63	31.6±8.57	1.589	0.115
Education	12.48±1.42	14.66±1.47	-7.560	**<0.001**
Abstinence (mo)	12.8 ± 12.4			
***Drug Dependence***				
Stimulants	50			
Nicotine	35	8		
Alcohol	35			
Opioids	16			
Cannabis	20			
Other	9			
***Drug Use Characteristics***				
Stimulant Use Onset Age (yrs)	17.3 ± 4.7			
Stimulant Use Duration (yrs)	15.7 ± 7.4			
***Behavioral Metrics***				
BIS	20.64±3.26	20.10±3.56	0.792	0.430
BAS	76.74±6.54	69.40±9.27	4.575	**<0.001**
Drive	12.80±2.17	10.34±2.26	5.551	**<0.001**
Fun Seeking	13.16±1.85	11.38±2.18	4.414	**<0.001**
Reward	17.40±1.94	17.10±1.75	0.812	0.419
BIS-11	72.16±11.54	57.30±6.71	7.871	**<0.001**
Motor	27.30±4.72	22.78±3.02	5.707	**<0.001**
Non-Planning	27.08±4.96	20.48±3.33	7.809	**<0.001**
Attentional	17.78±3.81	14.04±3.14	5.361	**<0.001**
PANAS-X				
Positive Affect	35.32±6.09	35.92±6.09	-0.488	0.626
Negative Affect	21.54±7.98	14.72±3.48	5.540	**<0.001**

Acronyms: *BAS*, Behavioral activation scale; *BIS*, Behavioral inhibition scale; *BIS-11*, Barratt impulsiveness scale version 11; *PANAS-X*, Positive and Negative Affect Schedule–Expanded Form.

### Structured Interviews and Questionnaires

#### Screening Assessment

All participants received structured interviews and behavioral measures. Drug dependence was assessed using the computerized Composite International Diagnostic Interview-Substance Abuse Module (CIDI-SAM) [21]. IQ was estimated with matrix and verbal reasoning Wechsler Abbreviated Scale of Intelligence subtests (WASI, Psychological Corporation, 1999) and was recorded to exclude subjects with low scores (IQ < 80). The Diagnostic Interview Schedule version IV is a computerized structured interview used to screen for psychiatric disorders. Participants completed this interview to exclude those with a history of psychiatric disorders as described above. Substance dependence severity was operationalized as the number of total substance dependence and abuse symptoms, quantified by the Diagnostic Interview Schedule version IV [22,23].

#### Behavioral Inhibition Scale (BIS) / Behavioral Activation Scale (BAS)

The Behavioral Inhibition and Activation Scale is a 20-item self-reported questionnaire used to measure responsiveness of motivational systems [24,25]. Behavioral approach and inhibition were operationalized as the total Behavioral Activation and Inhibition Scales, respectively.

#### Barratt Impulsiveness Scale (BIS-11)

This is a 30-item self-reported questionnaire used to measure impulsivity; participants rated whether phrases and words describing aspects of impulsivity were self-descriptive [26]. Impulsiveness was operationalized as the total Barratt score.

#### Positive and Negative Affect Scales

Positive and Negative Affect Schedule–Expanded Form (PANAS-X) quantifies a participant’s positive and negative affect using a series of 60 words and phrases that are rated on a scale of self-description [27]. Positive and negative affect were operationalized as the total PANAS-X score for positive and negative attributes.

### MRI Examination and Image Analysis

#### MRI Acquisition

Brain MRI was performed using a 3T MR scanner (General Electric, Milwaukee, Wisconsin) and standard quadrature head coil. Head motion was minimized using a VacFix head-conforming vacuum cushion (Par Scientific A/S, Odense, Denmark). Any subjects with ≥2 mm of head motion were excluded. High resolution T1-weighted SPGR-IR sequences (TR = 45ms, TE = 20ms, flip angle = 70°, 256 × 256 matrix, 240 × 240mm^2^ field-of-view (0.9 × 0.9mm^2^ in plane), 1.7mm slice thickness, and coronal plane acquisition) and resting-state functional scans (TR = 2000ms, TE = 30ms, flip angle = 30°, axial acquisition, 64 × 64 matrix, 3.4 mm × 3.4 mm in-plane voxel size, 3mm slice thickness, 1mm gap, 150 volumes) were acquired. During fMRI acquisition, participants were instructed to close their eyes, not think of anything in particular, and not fall asleep.

#### Image Preprocessing

**Fig 2:**
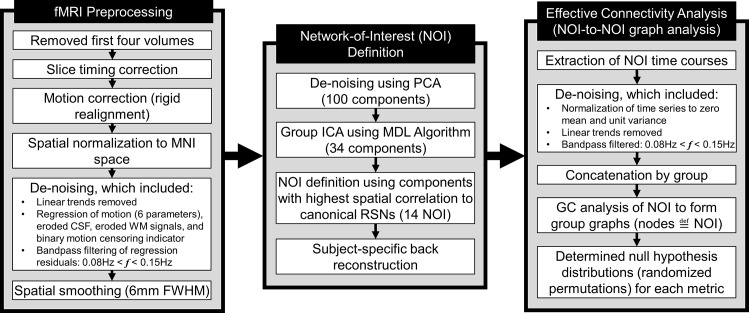
Diagram of the fMRI BOLD preprocessing, network-of-interest definition, and effective connectivity analysis pipelines.

Resting fMRI images were processed using the SPM8 toolbox in MATLAB. The first four volumes of each examination were excluded to avoid saturation effects (**Fig 2**). Standard pre-processing steps included slice timing correction, rigid realignment and motion correction (motion >1 voxel/TR was censored), spatial normalization, and de-noising. Motion parameters (three rotation and three translation parameters) for censorship were calculated for each time-point using corresponding SPM realignment pre-processing values. Anatomical volumes were segmented into gray matter, white matter, and CSF tissue maps, and the resulting binary masks were eroded (1 isotropic voxel) to mitigate partial volume effects. CSF and white matter time series were obtained using the mean signals from voxels based on eroded CSF and white matter SPM template masks. Mask erosion and time series extraction were performed using functions contained in the CONN toolbox [28]. After linear trends were removed, time series of the motion parameters, WM signal, and CSF signal were removed from the resting-state BOLD data using linear regression, and the resultant residual BOLD time series were band-pass filtered (0.008 Hz < ***f*** < 0.15 Hz) [29]. The resultant filtered time series were spatially smoothed with a 6mm full width at half maximum Gaussian kernel.

#### Networks-of-interest (NOI) definition and behavioral correlations

Group independent component analysis (ICA) was performed using the GIFT toolbox as previously reported in the literature [5,30–34] in order to define the networks-of-interest (NOI). For the purposes of this study, the term resting state networks (RSN) refers to the canonical spatial maps used to define the NOI. The term NOI refers to the independent components identified in our sample population and labeled by their corresponding RSN. The dimensionality of the data from each subject was first reduced to 100 components using principal component analysis. Subsequent group-level ICA yielded 34 components, the number of which was determined using the minimum description length (MDL) algorithm [35]. Fourteen canonical RSN templates (**Table 2**) were provided by Stanford's Functional Imaging in Neuropsychiatric Disorders (FIND) Laboratory [36]. At the group level, the 34 identified components were spatially correlated with the canonical RSN templates. Components with the highest spatial correlation to the canonical template were labeled with the corresponding standard RSN label. These labelled components formed the set of NOI for subsequent graph analysis. All components were visually inspected by a neuroradiology fellow (N.S.) and radiology resident (M.R.) independently to confirm accuracy with the canonical RSN templates. Concordance between inspectors was 100%. Subject-specific spatial maps and time courses were estimated using the GICA back-reconstruction function in GIFT.

**Table 2:** Canonical RSN included in the analysis and their corresponding symbol abbreviations. Spatial maps were provided by Stanford's Functional Imaging in Neuropsychiatric Disorders (FIND) Laboratory [36].

Resting State Network	Symbol
Auditory Network	AN
Anterior Salience Network	aSN
Basal Ganglia Network	BGN
Dorsal Default Mode Network	dDMN
High Visual Network	HVN
Left Executive Control Network	LECN
Language Network	LN
Precuneus Network	PCN
Posterior Salience Network	pSN
Primary Visual Network	PVN
Right Executive Control Network	RECN
Sensorimotor Network	SMN
Ventral Default Mode Network	vDMN
Visuospatial Network	VSN

For each subject, the strength (or coherence) of each NOI was operationalized as the mean beta value across spatial dimensions for that component in the mixing matrix. These values were regressed against impulsivity, approach, inhibition, and negative affect. Regressions between the NOI strength and subjects’ behavioral metrics were used to interpret the neuroimaging findings within the context of measurable behavioral characteristics.

#### Effective Connectivity Analysis

For each individual, the time courses corresponding to the NOI were obtained from the back-reconstruction function in group ICA. These NOI signals were linear trend removed, normalized to zero mean and unit variance, and band-pass filtered at 0.008–0.15 Hz. The resultant NOI time courses were temporally concatenated across individuals into SDI and control groups [37,38]. Effective connectivity between all 14 NOI time courses in the SDI and control graphs were calculated using Granger causality (GC) analysis implemented in the Granger Causal Connectivity Analysis (GCCA) MATLAB toolbox [20]. Significance was estimated by comparing observed group difference to a randomized null hypothesis distribution, and the test statistic was determined by the percentile position of the observed difference (i.e., the proportion of randomizations with values greater than or equal to the observed value). To determine null hypothesis distributions, subjects’ group labels were randomized and GC connectivity differences estimated for each randomization permutation until the aggregate randomization distribution achieved statistical stability. Significance level was α = 0.05 using false discovery rate (FDR) *q* < 0.05 to correct for multiple comparisons.

#### Global Network Measures

To provide global network measures, graph theory measures were used to describe the topology of the graphs of NOI. The purpose was to provide holistic, organ-level measures of brain connectivity for comparisons between groups. These measures included total weighted network density, local efficiency (derived from clustering coefficients), and global efficiency (derived from path lengths). These measures’ derivations and their justification have been previously described in detail [39]. Computational details are provided in **S1 Appendix**.

## Results

### Sample Population Demographics

There were no significant differences in age (*p* = 0.12) or sex (*p* = 0.87) between groups (**Table 1**). Years of education (*p*<0.01) and IQ (*p*<0.01) differed between groups. Controls had higher IQ and more years of education than SDI. All SDI met DSM-IV dependence criteria for stimulants. Drug use characteristics are also summarized in **Table 1**. Eight controls met dependence criteria for tobacco. No controls met dependence criteria for drugs or alcohol.

### Behavioral Metrics

Behavioral characteristics are summarized in **Table 1**. No group difference in BIS inhibition was observed (*p* = 0.43). A significant group difference in behavioral approach was observed, with SDI exhibiting higher total BAS scores than controls (*p*<0.001). Further analysis of BAS subscales showed that SDI had higher scores on “drive” (*p*<0.001) and “fun-seeking” (*p*<0.001), but not “reward-responsiveness” (*p* = 0.42). As expected, SDI reported higher impulsivity than controls (*p*<0.001) as well as significant differences in the motor, non-planning, and attentional subscales (*p*<0.001). No significant difference in positive affect was observed (*p* = 0.626); however, a large difference in negative affect was observed (*p*<0.001), with SDI demonstrating greater negative affect scores than controls.

### Network Analysis

#### Directed Connectivity Analysis

**Fig 3:**
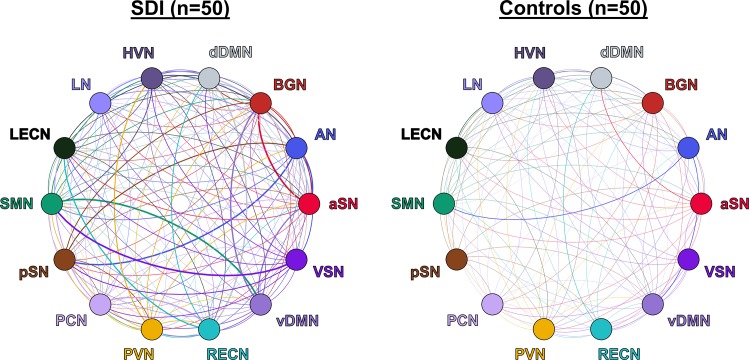
Effective connectivity network density graphs of SDI and controls. Thickness of each line corresponds to the Granger causal strength and color corresponds to the efferent network. SDI network density was significantly greater than healthy controls (*p*<0.001).

**Fig 4:**
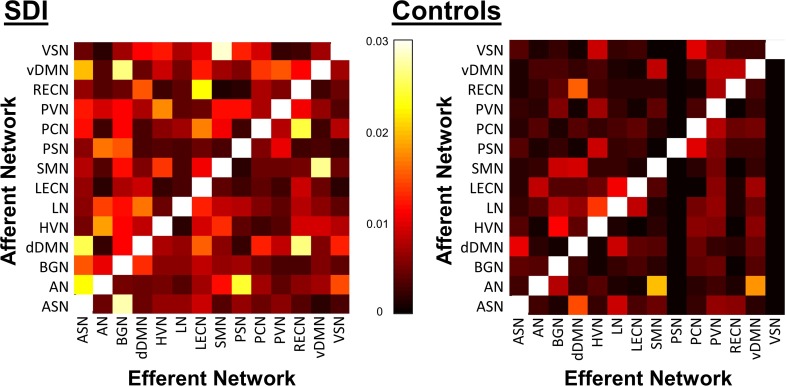
Directed GC matrices for SDI (*left*) and controls (*right*). Colorbar corresponds to logarithm of F values.

**Fig 5:**
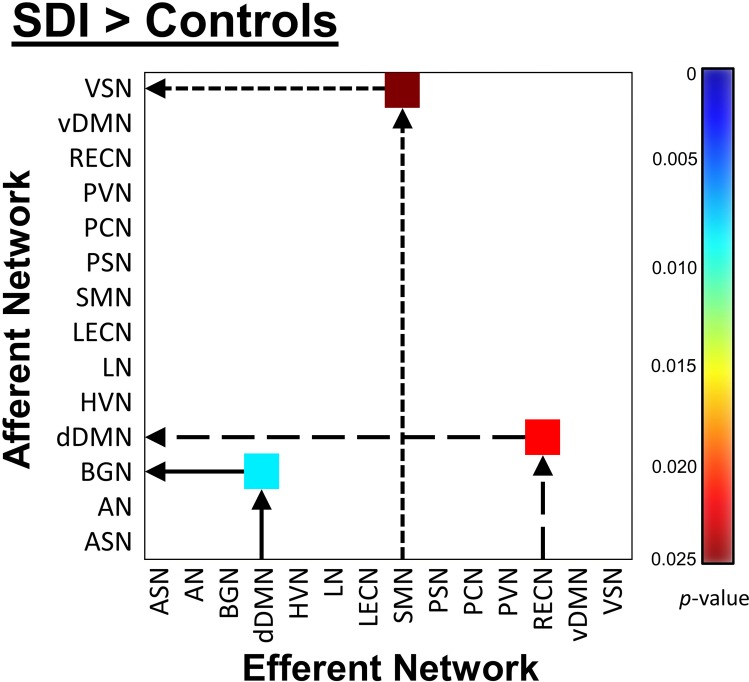
Effective connectivity matrix illustrating the FDR-corrected group differences between SDI and healthy controls. Colorbar corresponds to *p*-values (white represents non-significance). Arrows indicate Granger causal direction.

**Fig 6:**
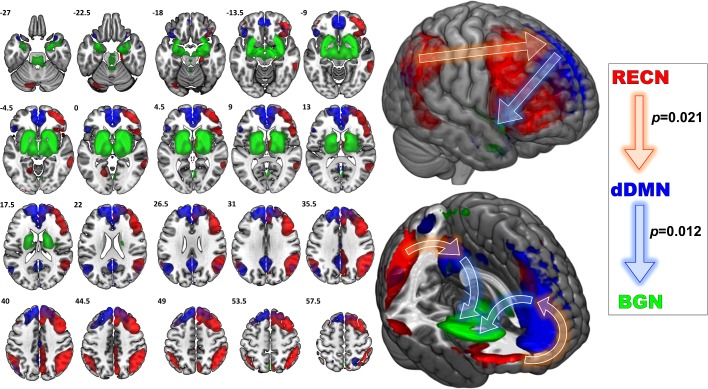
*Left*, NOI demonstrating significantly greater Granger causal relationships in SDI compared to controls. Illustrated are the NOI relationships that differed between groups; colors correspond to NOI labels on right. Printed numbers in upper left corner of each slice correspond to Z-axis coordinates in MNI space. *Right*, Granger causal relationships demonstrating greater directed information flow in SDI compared to controls (FDR corrected). *RECN*, right executive control network; *dDMN*, dorsal default mode network; *BGN*, basal ganglia network. Values indicate Granger causal *p*-values. *Middle*, whole brain illustrations of NOI identified. Arrows reflect Granger causal influence.

SDI network density was significantly greater compared to controls (*p*<0.001, **Fig 3**). This measure reflects increased overall mean GC causal connectivity strength between all 14 NOI compared to controls. Specifically, GC analysis results show that among 182 possible between-network pairs (**Fig 4**), only three pairs differed significantly across group in the FDR corrected data (**Fig 5**). Compared to controls, SDI showed stronger effective connectivity from the RECN to the dDMN and from the dDMN to the BGN (**Fig 6**). In addition, SDI showed stronger effective connectivity from the SMN to the VSN. SDI showed greater bidirectional connectivity (reciprocal GC connections) whereas controls showed more unidirectional connectivity among the 14 network components.

#### Network-Behavioral Correlations

**Fig 7:**
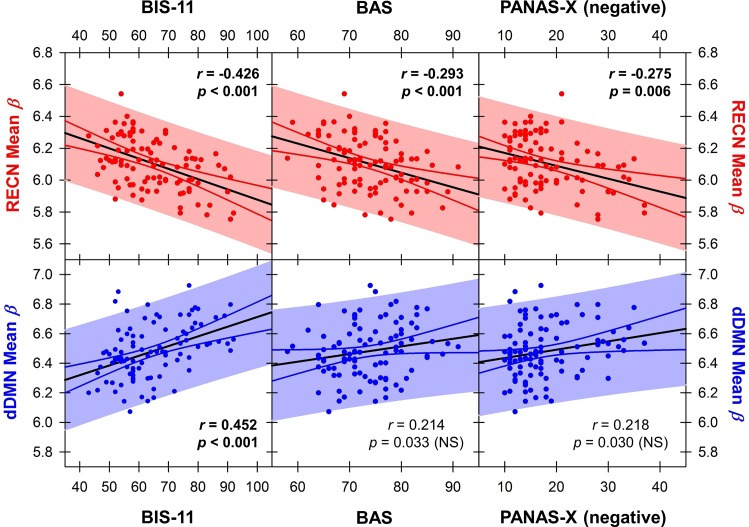
Correlations between mean beta value within the RECN and dDMN with impulsivity, approach, and negative affect metrics. Solid black lines indicate the linear regression, solid colored lines indicate the 95% confidence interval, and colored shaded regions indicate the prediction interval. Each point reflects a single participant’s mean beta within a given network, and their score on the given behavioral metric. Bolded text indicates statistically significant pairwise correlations using the Bonferroni method to correct for eight multiple comparisons. *NS*, not statistically significant.

The strength of the RECN correlated negatively with impulsivity (p<0.001), behavioral approach (p<0.001), and negative affect across the population (p = 0.006) (**Fig 7**). In contrast, the strength of the dDMN correlated positively with impulsivity (p<0.001), but not behavioral approach (p = 0.034) or negative affect (p = 0.030) after correcting for multiple comparisons (**Fig 7**). No NOI correlated with positive affect or educational attainment in years.

#### Global Graph Measures

**Fig 8:**
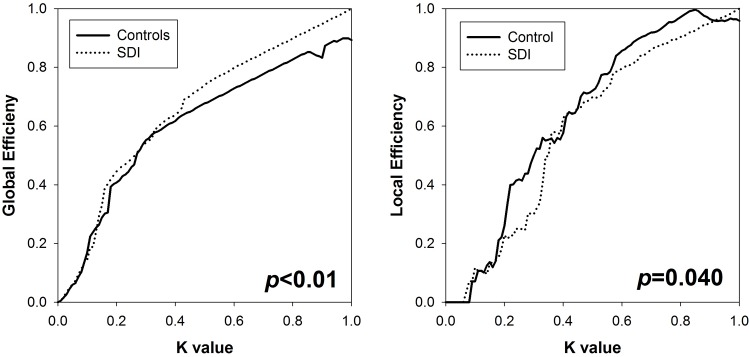
Global efficiency (*left*) and local efficiency (*right*) in SDI and controls as a function of network cost.

Group comparison results for local and global efficiency across the domain of cost functions are illustrated in **Fig 8**. Global efficiency was significantly higher in SDI than in controls (p<0.01), suggesting greater global integration. Local efficiency was higher in controls (p<0.05), suggesting greater local specialization. These findings in conjunction suggest reduced small-worldness in SDI compared to healthy controls.

## Discussion

This study revealed greater effective connectivity network patterns in abstinent substance dependent individuals compared to healthy controls. Specifically, in drug users who have been abstinent for on average over one year, effective connectivity analysis revealed increased information flow from the RECN to dDMN and dDMN to BGN compared to controls. The areas of increased effective connectivity observed in our study correspond to regions involved in executive control (RECN), interoception (dDMN), reward (BGN), and habit (BGN). The mean strength of the RECN component correlated negatively with impulsivity, behavioral approach, and negative affect. In contrast, the mean strength of the dDMN component correlated positively with impulsivity and trended towards positive correlations with behavioral approach and negative affect. Given the prolonged abstinence of our SDI sample population, these findings are consistent with the hypothesis that successful long-term abstinence is associated with increased top-down cognitive control.

### Increased Effective Connectivity from RECN to dDMN

The pattern of increased effective connectivity from RECN to dDMN is consistent with increased top-down executive control in long-term abstinence. Previous work has demonstrated both task-related and resting state hyperactivity within executive control and default mode cortices in abstinent stimulant dependence associated with heightened behavioral monitoring [17,40,41]; however, this is the first study to suggest that these neural signals follow a directional flow of information from RECN to dDMN. Connolly et al. [40] conducted a cognitive control task-based fMRI study of short- (2.4 ± 1.34 weeks) and long-term (69 ± 17.49 weeks) abstinent cocaine addicts. Abstinent cocaine users demonstrated increased activity in PFC, cingulate, and inferior frontal gyri compared to healthy controls. Moreover, short-term abstinent individuals showed right dorsolateral PFC (corresponding to RECN in our study) hyperactivity positively correlating with inhibitory control. Long-term abstinent individuals showed the same finding as well as anterior and mid cingulate (corresponding to part of the dDMN in our study) hyperactivity positively correlating with cognitive errors and heightened behavioral monitoring in abstinence. The present study showed that the RECN strength was negatively correlated with subjects’ impulsiveness, while dDMN strength was positively correlated with impulsiveness. Our results advance our understanding of neural network changes during substance use disorder remission: as abstinence progresses, cortices within the RECN and dDMN may become hyperactive to exert top-down executive control in a directed fashion; this neuroadaptive change may be associated with decreases in impulsivity and increases in inhibitory control.

However, the top-down cognitive control hypothesis is not straightforward because in addition to executive function and cognitive control, affect plays an important role. Albein-Urios et al. [42] showed that short-term abstinent (2.5 ± 5.5 months) cocaine dependent individuals had increased right dorsolateral PFC and bilateral temporoparietal cortex activation during negative emotion experiences without a concomitant increase in the subjective negative experience itself, suggesting an exaggerated neural response in these regions is required to produce normal levels of emotional salience. The regions reported closely resemble by visual comparison the RECN identified by our analysis. Albein-Urios et al. posited that these areas demonstrate increased sensitization toward negative emotions in SDI. If increased RECN top-down control is a durable feature of long-term abstinence, the literature thus far suggests that its manifestations in human behavior are complex and not reducible to a single neuropsychological construct. Our finding that RECN strength is negatively correlated to negative affect while dDMN strength trended towards positive correlation with negative affect provides further evidence that the top-down control model may involve affective components as well, possibly through reciprocal connectivity with limbic areas.

Right-sided lateralization of our ECN findings is not unexpected given the asymmetric functional specialization of cerebral hemispheres in healthy humans. Cocaine dependent patients exhibit reduced resting state interhemispheric connectivity compared to healthy controls in prefrontal and parietal cortices [43], suggesting increased lateralization of function. Connolly et al. [40] reported that hyperactivity in the inferior frontal gyrus correlated with inhibitory control was greater in the left hemisphere in short-term abstinent individuals and greater in the right hemisphere in long-term abstinent individuals. They hypothesized that a shift from left to right inferior frontal gyrus for inhibitory control may reflect a transition from short-term to long-term abstinence. Our results of increased RECN effective connectivity in long-term abstinent stimulant dependence are consistent with this hypothesis, although future longitudinal studies are required for substantiation.

### Increased Effective Connectivity from dDMN to BGN

Although the DMN is incompletely understood, growing evidence demonstrates its roles in internally directed tasks such as spontaneous cognition [44], self-referential [45] and autobiographical [46] thought, and social understanding of others [47]. We demonstrated increased effective connectivity from the dDMN to BGN; however, this finding must be interpreted in the context of the structures within the NOI identified as BGN (**Fig 5**). This NOI included basal ganglia, thalamus, amygdala, hippocampus, hypothalamus, midbrain, and pons. Thus, BGN included several key regions of the bottom-up mesocorticolimbic circuit including the ventral tegmental area, nucleus accumbens, amygdala, and striatum.

Prior studies have demonstrated hypoactivity in stimulant users in the dDMN and in BGN as well as decreased connectivity between these networks. In active cocaine users, Tomasi et al. [48] showed that cocaine cues disengaged fMRI activity in the ventral striatum, hypothalamus, and DMN in proportion to density of striatal dopamine receptors by PET. DMN activation has been shown to predict performance errors, is diminished in active cocaine dependence, and the extent of altered error-preceding activation has been reported to correlate with years of cocaine use [49]. Gu et al. [15] used a seed-based fMRI paradigm in active cocaine users and found significantly decreased functional connectivity between multiple regions of the DMN and BGN. McHugh et al. [50] showed that individuals successfully abstinent 30 days after detoxification had stronger functional connectivity between the amygdala, ventromedial PFC, and anterior cingulate cortex compared to those who had relapsed. By visual comparison, these regions correspond to structures within the NOI identified as dDMN and BGN in our study. Connolly et al. [40] demonstrated increased anterior and mid cingulate activity in long-term abstinent compared to short-term abstinent individuals, activity which correlated with heightened behavioral monitoring. Together, these prior studies suggest that DMN activity may change during abstinence. Initial hypoactivation during early abstinence may transition to hyperactivation and increased connectivity with long-term abstinence. One interpretation is that findings of increased effective connectivity from dDMN to BGN in long-term abstinence may be a compensatory mechanism related to behavioral monitoring not seen in active users. However, longitudinal studies are needed to demonstrate this.

### Increased Global and Decreased Local Integration

Our findings of increased bidirectional connectivity, increased global efficiency, and decreased local efficiency in long-term abstinent SDI compared to healthy controls suggests pathologically greater global integration and lower local integration in SDI; that is, a connectomic decrease in small-worldness. Similar findings in humans have only been reported using EEG data in 1–3 week abstinent methamphetamine dependent persons. Ahmadlou et al. [51] showed that these patients demonstrated a deviation from small-worldness and increased global hypersynchronization in the gamma frequency band, the EEG band most reactive to cognitive information processing. In contrast, active cocaine users demonstrated less global connectivity compared to healthy controls during a Stroop task; however, after adjusting for individual connectivity, cocaine dependent individuals showed greater intrinsic connectivity in the ventral striatum, putamen, inferior frontal gyrus, anterior insula, thalamus and substantia nigra [52].

Several animal studies provide important context for the interpretation of our findings. Schwarz et al. [53] used a pharmacological challenge design which revealed that rats under the acute effects of amphetamine compared to a saline vehicle exhibited less clustering (small-worldness) and increased connectedness within somatosensory, motor, cingulate, prefrontal, and insular cortices. In the rhesus monkey model, active cocaine self-administration was associated with decreased global functional connectivity that selectively affected top-down prefrontal circuits and control behavior while sparing limbic and striatal areas [54]. Interestingly, impaired connectivity between prefrontal and striatal areas during abstinence predicted cocaine intake when these monkeys were again provided access to cocaine (i.e., prediction of relapse), consistent with the connectivity pattern associated with relapse in humans as reported by Camchong et al. [18].

Together these findings suggest there is globally decreased connectivity in active users and short-term abstinent with a transition to globally increased connectivity in long-term abstinent users. These findings may improve clinical management if global connectivity patterns can be used to predict abstinence success or trajectory in humans. Future longitudinal studies comparing global connectivity in active, short-term, and long-term abstinent drug users must be performed to address this question. Another approach could involve correlating abstinence duration with global connectivity across individuals, an approach we could not implement due to the group-level nature of our statistical design.

### Limitations

#### Controversy surrounding Granger Causality

While our study provides several important novel findings, it has limitations. Influences between specialized neural systems exist on a spectrum of temporal lag. Functional connectivity using temporal correlation reflect influences with causal latencies that are below the temporal resolution of the repetition time. These influences are not truly contemporaneous *in vivo*, but appear so by fMRI as a result of low temporal sampling and temporal blurring induced by the hemodynamic response function. Time-lag based measures such as Granger causality reflect slower influences with greater causal latencies that occur on the order of hundreds of milliseconds, which may provide greater power in predicting cause-effect relationships at the timescale of conscious thought [55].

Neural signals between two nodes may have significantly different physiologic functions depending upon the directionality. As a result, segregating neural influences according to their directionality is necessary in order to properly examine brain function. Methods of examining effective connectivity using fMRI data include structural equation modelling [56] and dynamic causal modelling [57]. These methods require *a priori* hypotheses describing the theoretical connectivity structure and are limited to models consisting of a small number of nodes. We used an alternative method, Granger causality, which is based on time-lag regressions and is more data-driven.

Granger causality is increasingly used in fMRI-based neuroscience [58–62] and has been previously applied specifically to independent component analysis as in our study [37,63–70]. However, criticisms of the application of Granger causality to fMRI data have included [71]: (1) lack of evidence that Granger causality in fMRI-level time series reflects causality in neuronal-level time series, (2) insufficient temporal sampling relative to the timescale of neuronal events, and (3) the possibility that spurious findings may result from systematic differences in hemodynamic response functions. Several recent developments have provided evidence that fMRI Granger causality reliably reflects neuronal causality [60,71–73]. Seth and colleagues [74] demonstrated that Granger causality is reliably invariant to inter-regional differences in the hemodynamic response function, including the time-to-peak. However, they reported significant effects of temporal resolution on their results. Wen and colleagues [60] demonstrated that fMRI-based Granger causality is a monotonic function of neural Granger causality. Importantly, they showed that this relationship can be reliably detected using conventional fMRI temporal resolution and noise levels as was used here. However, they cautioned that differences in the hemodynamic response could lead to spurious results.

The impact of hemodynamic response variability is currently debated. Schippers and colleagues [72] demonstrated that hemodynamic response variability was minimized by multisubject group inference. Statistically, this is intuitive because population averaging will augment systematic differences (e.g., true neuronal differences) while suppressing random or pseudorandom differences (e.g., hemodynamic response variability). Some authors speculate that HRF variability could be systematic [75], and indeed this is a confound that by design exists in the majority of between-group fMRI studies using independent samples [76–78]. Accordingly, we cannot exclude that systematic differences in the neurovascular response to neural activity between groups may have contributed to our findings.

#### Other Limitations

Network resolution was limited by the manner in which independent component analysis identifies temporally coherent signals across the brain. For example, the network component identified as BGN included several non-basal ganglia structures, such as the thalamus, amygdala, hippocampus, and midbrain. Additionally, concatenation across individuals precluded correlation of individual psychological measures to resting state network Granger causality; as such, correlations between the strength of each NOI and behavioral metrics were used to provide psychological context for the findings. Lastly, polysubstance use and low educational attainment among psychostimulant users may be viewed as potential confounds or representation of real world clinical features. There is significant literature describing the correlation between drug use and low educational attainment; it is debated whether low educational attainment is the cause or result of drug use disorders [79–81]. More recently, however, authors have reported that this correlation is due in part to shared genetic factors [82] while others report that it is due to shared environmental or non-genetic familial risk factors [83,84]. These studies suggest that low educational attainment is a behavioral component of the pathology of substance use disorders. With regard to polysubstance use among SDI, while this limitation prevents our findings from being attributed to a single drug, it strengthens our results by providing biological and ecological validity. Epidemiologic studies have demonstrated that psychostimulant dependence does not naturally occur in isolation; rather, most patients meet dependence criteria for other drugs of abuse [85,86]. Our sample population thus reflects the real-world, clinical population of patients with stimulant dependence.

## Conclusion

Increased effective connectivity in long-term abstinent drug users may reflect improved cognitive control and behavioral monitoring (ECN) over self-referential thought (DMN), habit (BGN), and reward (BGN) processes in long-term abstinent drug users. Higher global and lower local efficiency across all networks in SDI compared to healthy controls may reflect connectivity changes associated with drug dependence or remission. Future, longitudinal studies are necessary to definitively characterize connectomic changes across the natural history of substance use disorders.

## Supporting Information

S1 Appendix Graph theory metrics computation. (DOCX)
