# Supplementary material for: Top-Down Network Effective Connectivity in Abstinent Substance Dependent Individuals
Source: PLoS One. 2016 Oct 24;11(10):e0164818. doi: 10.1371/journal.pone.0164818 (PMC5077096; doi:10.1371/journal.pone.0164818)
Supplement: S1 Appendix — (DOCX) [file pone.0164818.s001.docx]

**S1 Appendix. Graph theory metric computation.**

To compute graph theory metrics the GC connectivity matrices were converted to a binary directed adjacency matrix, where connectivity strengths above and below a certain threshold cost level are set to 1 and 0, respectively (1). The cost threshold, $K(\text{G}_{x})$, was calculated by first sorting all elements other than the auto-correlating diagonals (identity axis) of the connectivity matrix in descending rank and keeping only the top x% (sub-graph, $\text{G}_{x}$). Cost was then computed as the fraction of highest strength edges above the given threshold divided by the total number of edges:

$$K(\text{G}_{x})\equiv\frac{\left| \mathcal{E}\left( \text{G}_{x} \right) \right|}{\left| \mathcal{E}\left( \text{G}^{Sat} \right) \right|}$$

where $\text{G}^{Sat}$ represents an edge-saturated network with the same $\mathcal{N}$ and the function $\left| \mathcal{E}\left( \text{G} \right) \right|$ represents the cardinality of $\mathcal{E}\left( \text{G} \right)$. Therefore, a low value reflects a sparse network. The Brain Connectivity Toolbox (2) was used to calculate global and local efficiency at each cost level. Since there was no *a priori* reason to select a particular network cost threshold, connectivity metrics as a function of cost were computed and integrated across the cost domain [0, 1]: $\int_{0}^{1} K\left( \text{G}_{x} \right)dx$ (1). This approach abides by prior methodological recommendations to separate network cost from network topology (1). To test for significant differences between groups, a non-parametric randomized permutation test was performed. Subjects’ group labels were randomized and graph theory measures were calculated for each randomization permutation until the randomization distribution demonstrated statistical stability. These distributions formed the null hypothesis distributions representing no group differences for total weighted network density, integrated global efficiency, and integrated local efficiency. Significance of groups differences for each metric was determined by the percentile position within the null hypothesis distribution.

**References:**

1. Ginestet CE, Nichols TE, Bullmore ET, Simmons A. Brain network analysis: separating cost from topology using cost-integration. PloS One. 2011;6(7):e21570.

2. Rubinov M, Sporns O. Complex network measures of brain connectivity: uses and interpretations. NeuroImage. 2010 Sep;52(3):1059–69.
